# Using Patient-Specific 3D Modeling and Simulations to Optimize Microwave Ablation Therapy for Liver Cancer

**DOI:** 10.3390/cancers16112095

**Published:** 2024-05-31

**Authors:** Amirreza Heshmat, Caleb S. O’Connor, Jessica Albuquerque Marques Silva, Iwan Paolucci, Aaron Kyle Jones, Bruno C. Odisio, Kristy K. Brock

**Affiliations:** 1 Department of Imaging Physics, The University of Texas MD Anderson Cancer Center, 1515 Holcombe Blvd., Houston, TX 77030, USA; csoconnor@mdanderson.org (C.S.O.); kyle.jones@mdanderson.org (A.K.J.); kkbrock@mdanderson.org (K.K.B.); 2 Department of Interventional Radiology, The University of Texas MD Anderson Cancer Center, 1515 Holcombe Blvd., Houston, TX 77030, USA; jalbuquerque@mdanderson.org (J.A.M.S.); ipaolucci@mdanderson.org (I.P.); bcodisio@mdanderson.org (B.C.O.)

**Keywords:** microwave ablation, liver cancer, finite element method, image-guided cancer therapy, necrotic tissue, 3D model, ablation zone, thermal ablation

## Abstract

**Simple Summary:**

Microwave ablation (MWA) is a minimally invasive image-guided approach that uses microwaves emitted by an antenna to heat and destroy cancer cells. MWA is commonly used for treating liver tumors due to its efficient heating, minimal susceptibility to heat sink effects, and ability to treat larger tumors effectively. However, achieving effective ablation, defined by a minimal ablative margin of at least 5 mm, is crucial to prevent tumor recurrence and is affected by factors like tumor size and location, antenna placement, patient health status, and MWA device parameters. Challenges in antenna placement and the need for precise monitoring during MWA therapy often result in repeated adjustments and potential over-ablation, risking damage to healthy tissues. This study demonstrated that patient-specific three-dimensional models in MWA therapy can accurately predict the delivered ablation zone and have the potential to improve treatment efficacy, enabling more precise ablations and better patient outcomes with minimal unnecessary tissue damage and adequate margins.

**Abstract:**

Microwave ablation (MWA) of liver tumors presents challenges like under- and over-ablation, potentially leading to inadequate tumor destruction and damage to healthy tissue. This study aims to develop personalized three-dimensional (3D) models to simulate MWA for liver tumors, incorporating patient-specific characteristics. The primary objective is to validate the predicted ablation zones compared to clinical outcomes, offering insights into MWA before therapy to facilitate accurate treatment planning. Contrast-enhanced CT images from three patients were used to create 3D models. The simulations used coupled electromagnetic wave propagation and bioheat transfer to estimate the temperature distribution, predicting tumor destruction and ablation margins. The findings indicate that prolonged ablation does not significantly improve tumor destruction once an adequate margin is achieved, although it increases tissue damage. There was a substantial overlap between the clinical ablation zones and the predicted ablation zones. For patient 1, the Dice score was 0.73, indicating high accuracy, with a sensitivity of 0.72 and a specificity of 0.76. For patient 2, the Dice score was 0.86, with a sensitivity of 0.79 and a specificity of 0.96. For patient 3, the Dice score was 0.8, with a sensitivity of 0.85 and a specificity of 0.74. Patient-specific 3D models demonstrate potential in accurately predicting ablation zones and optimizing MWA treatment strategies.

## 1. Introduction

Thermal ablation modalities such as radiofrequency, microwave, and cryoablation have been widely utilized in clinical practice to treat various types of primary and secondary cancers in various organs. Particularly for primary and secondary liver cancer, microwave ablation (MWA) has been established as the energy modality of choice among several due to its improved heating profile, less susceptibility to heat sink effects, safety profile, shorter ablation duration, and expanded ablation zone; it can also treat larger tumors and achieve higher temperatures [1,2].

Several studies have highlighted that MWA achieves higher temperatures up to 152 °C compared to 106 °C for radiofrequency ablation and generates larger ablation zones [3,4,5,6]. The MWA ablation volume is approximately 33% larger than the radiofrequency ablation volume [2], and another study reported that the MWA ablation volume is more than double that of the radiofrequency [3]. Violi et al. [7] reported two-year local tumor progression rates of 6% for MWA and 12% for radiofrequency ablation. In addition, several studies reported up to 45 % lower local tumor progression rates after MWA treatment than radiofrequency [2,3]. A retrospective study involving 250 patients with 435 tumors treated by MWA and 53 patients with 75 tumors treated by laser ablation reported higher overall survival rates at 1, 3, and 5 years for MWA (94.3%, 65.4%, and 49.1%, respectively) compared to those for laser ablation (96.2%, 54.7%, and 30.2%). In addition, MWA demonstrated a significantly lower complication rate of 2.9% versus 7.9% for laser ablation, indicating enhanced safety and greater efficacy in patient outcomes [8]. These findings suggest that MWA is preferable to laser ablation when treating hepatocellular carcinoma.

Recent advancements in thermal ablation have focused on optimizing treatment efficacy and safety. Studies have indicated that pulsating heat can reduce maximum temperatures by up to 30%, diminish risks such as steam popping during thermoablation, and achieve comparable tumor necrosis to constant heat sources [9]. Furthermore, using multiple antennas has created more extensive ablation zones than a single antenna, even when the total power and energy remain consistent across all cases; employing multiple antennas rather than a single antenna offers the advantage of forming larger ablation zones. In addition, configurations with multiple antennas consistently achieve lower peak tissue temperatures than those with a single antenna [10].

MWA is a minimally invasive image-guided technique where an antenna, placed near or within a tumor, emits microwaves to heat and destroy the surrounding tissue through cellular denaturation and coagulative necrosis. MWA for malignant liver tumors demonstrates an increased success rate when combined with chemotherapy and radiotherapy [1,11]. A key benefit of MWA is its ability to enhance local tumor control and prolong progression-free survival in patients.

It is crucial to ensure complete tumor destruction and adequate ablation margins to prevent local tumor progression following thermal ablation effectively. Kei et al. [12] first demonstrated the critical importance of this relationship, which has been consistently confirmed by subsequent studies [13,14,15,16,17,18,19,20,21]. It is widely accepted that ensuring ablation margins of at least 5 mm is a critical factor in achieving local tumor control post-ablation. Achieving comprehensive ablation coverage with a minimal ablative margin (MAM) of ≥5 mm is crucial for achieving effective treatment outcomes [17,22,23], as not attaining such MAM is linked to local recurrence [1,2,24]. Reaching the MAM is challenging, and it depends on several factors, including tumor morphology and location, antenna positioning, treatment duration, the delivered energy quantity, the number of ablation cycles, and the antenna type [2,25,26]. In addition, antenna positioning is performed manually, with computed tomography (CT) guidance, and inaccurate or suboptimal placement of the antenna on the first attempt necessitates its repositioning, sometimes several times. Moreover, continuously monitoring MWA procedures to assess tissue damage and tumor destruction and to determine whether the MAM has been achieved is technically and clinically highly challenging, leading to additional re-ablation cycles during single sessions and over-ablation, potentially causing unnecessary irreversible damage to healthy tissue.

Leveraging patient-specific 3D models with computer simulations has shown promise in improving outcomes across various minimally invasive therapies [27,28,29,30,31]. Such models have been implemented to improve tumor visibility, optimize antenna placement, and tailor treatment parameters like input power and ablation time to the individual characteristics of each patient. It has been successfully adapted to other minimally invasive treatments, such as radiofrequency and laser ablation, demonstrating its effectiveness. Developing customizing 3D models for individual MWA treatments can potentially reduce the need for re-ablation cycles and prevent over-ablation, thereby improving the overall treatment efficacy. In this study, we used clinical ablation image data and finite element techniques to develop and validate patient-specific 3D models that incorporate clinical parameters to simulate MWA procedures with the primary objective of predicting the extent of the ablation zone before initiating therapy. Integrating these models and computational simulations could optimize treatment strategies and improve progression-free and overall survival rates for liver cancer patients.

## 2. Materials and Methods

### 2.1. Patient Characteristics, Ablation Procedure, and Ablation Margins Assessment

This is a retrospective study, HIPPA-compliant, with a waiver of informed consent approved by our institutional board review via our prospectively maintained liver ablation registry. The methodology involved developing patient-specific 3D models using retrospective clinical image data from patients who had a single ablation probe, full liver CT obtained with the probe inserted, and no intra-procedure probe adjustment. This retrospective data set was selected from patients treated at our institution between mid-June 2020 and early October 2023. Three patients met these criteria and were included in the study (Table 1). Their selection was based on the availability of complete clinical data and imaging required for accurate 3D model reconstruction. The ablation procedures were performed per our standard of care, as previously described [23]. In short, ablations were performed under general anesthesia support by a board-certified interventional radiologist with 15 years of experience. Intravenous contrast media-enhanced CT was performed before and after ablation delivery for procedure planning and ablative margins assessment, respectively. Microwave antenna placement was performed by manual correction under intermittent CT image guidance. MAMs were evaluated using our novel ablation confirmation methodology intraprocedural [32]. The ablation confirmation uses Morfeus [33], a deformable image registration algorithm, to confirm ablation coverage. This technique involves deforming the pre-operative liver surface to match the intra-operative liver surface, facilitating the accurate mapping of the tumor onto the ablation zones. This allows for precise margin assessment, enabling clinicians to evaluate the adequacy of the ablation.

### 2.2. Model Construction

Simulations considered the clinical parameters, including input power, ablation time, and microwave antenna insertion. Customized input power levels were applied with a triaxial microwave antenna at 2.45 GHz (Neuwave, Ethicon), and the simulations were conducted using COMSOL Multiphysics (v6.0), MATLAB (R2022b), and Python (v3.9). Using the finite element method, the model considers coupled electromagnetic wave propagation and bioheat transfer for estimating temperature distribution in biological tissue, tumor destruction, and tissue damage during MWA over time. The 3D model considers various factors, including how electromagnetic waves travel through tissues, how heat is transferred, and how tissues respond to this process over time.

**Figure 1:**
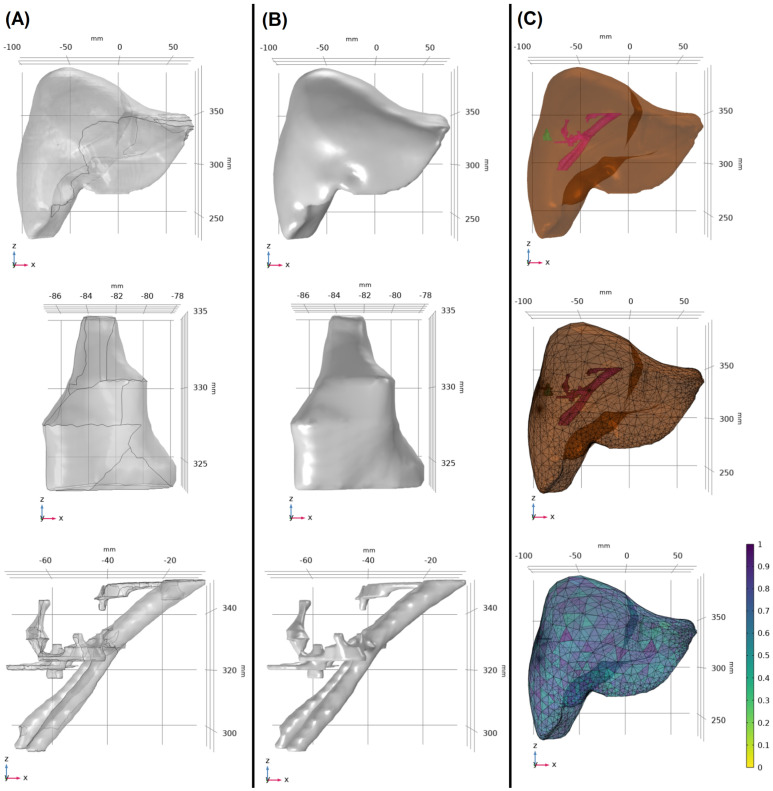
(**A**) Surface segmentation of the liver, tumor, and blood vessels for patient 1. (**B**) All components were modified and converted to 3D solid volume models. (**C**) The patient-specific 3D model was created (**top**), and the appropriate mesh volume was applied to the model (**middle**). The mesh test was applied to control the mesh quality, and the mesh size was modified accordingly (**bottom**).

**Figure 2:**
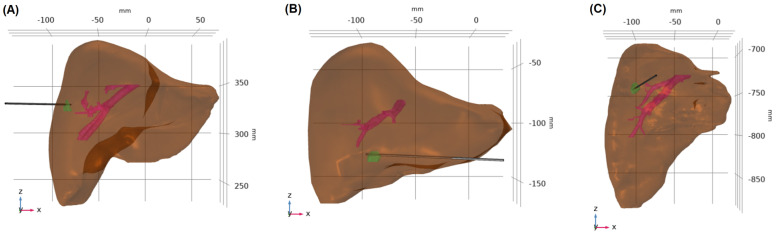
Microwave antenna insertion in patients. This figure illustrates the positioning of the microwave antenna for microwave ablation treatments in three different patients: (**A**) patient 1, (**B**) patient 2, and (**C**) patient 3.

Data collection involved obtaining contrast-enhanced CT images with the clear visualization of several anatomical features, including liver, tumor, blood vessels, ablation zone, and antenna insertion path, from three patients who underwent MWA treatment. These three cases were specifically selected because they each had a final CT scan available that covered the complete extent of the liver, the approximate ablation time was recorded (10 min ± 30 s), and no antenna repositioning was required during the ablation procedure. Segmentation techniques were used to extract the regions of interest, providing surface data for livers, tumors, and blood vessels (Figure 1A). For the segmentation of biological tissues, initial contours are generated using the nnU-Net, an artificial intelligence model tailored for medical image segmentation. These contours are reviewed and verified by a board-certified radiologist to ensure accuracy and clinical relevance. Any required edits to the contours are performed using a treatment planning system (RayStation, RaySearch Laboratories, Stockholm, Sweden). Once finalized, these segmented images are imported into COMSOL for modeling and simulation processes. These segmented data were modified and converted into 3D volumetric solid models (Figure 1B), resulting in unique 3D models for each patient (Figure 1C). In addition, Figure 2 demonstrates that the placement of the microwave antenna closely mimics clinical microwave antenna insertion for all three patients.

In this study, the distances between the tumors and the liver surface and from the tumors to the nearest major blood vessels for each patient were measured. For patient 1, the tumor is located 0.1 mm from the liver surface and 12.4 mm from the nearest major blood vessel. For patient 2, the tumor is 0.2 mm from the liver surface and 24.4 mm from the nearest vessel. For patient 3, the distances are 9.1 mm to the liver surface and 19.0 mm to the nearest major blood vessel. In addition, the volumes of the tumors, blood vessels, and livers for all three patients are described in Table 1.

Vendor-based ablation zone predictions (Vendor Prediction) were created in 3D based on the provided information, including length, diameter, and distal burn characteristics at 10 min for input powers of 65 W and 50 W. The 3D ellipse based on the vendor data was placed along the axes of the antenna at the distal burn location. This approach allowed us to compare the Vendor Prediction with the clinical data and patient-specific 3D models.

### 2.3. Mathematical Model

The 3D finite element method in COMSOL was used to simulate MWA by solving coupled equations related to the electromagnetic field and heat transfer in biological tissue. The propagation of the microwave field generated by the antenna is expressly governed by a form of Maxwell’s equation:

(1) $$\nabla \times {\mu_{r}}^{-1}\left(\nabla \times E\right)-k_{0}^{2}\left(\varepsilon_{r}-\frac{j\sigma }{\omega \varepsilon_{0}}\right)E=0$$

where E is the electric field (V/m) generated by the microwave antenna, $\mu_{r}$ is the relative permeability, and $k_{0}$ is the propagation constant in free space (1/m), defined as $\frac{2\pi }{\lambda }$, where λ is the wavelength (m). $\varepsilon_{r}$ and σ are the relative permittivity and electrical conductivity (S/m), respectively, of tissue. In addition, ω is the angular frequency (rad/s), defined as 2πf, where f is the frequency of an electromagnetic wave (Hz), and $\varepsilon_{0}$ is the relative permittivity in free space (8.854 $\times \;10^{-12}$ F/m).

MWA raises the tissue temperature, causing water evaporation and structural changes that alter tissue proteins. These alterations influence the tissue’s conductivity, permittivity, and response to electric fields. Changes in these electrical and thermal properties directly impact the tissue’s other characteristics and affect the electromagnetic energy distribution. Therefore, temperature-dependent electrical conductivity and permittivity of the liver and tumor were included in the calculations [34,35].

(2) $$\varepsilon_{r}\left(T\right)=l_{3}\left[1-\frac{1}{1+exp[l_{1}(\;l_{2}-T)]}\right]+1$$

(3) $$\sigma \left(T\right)=c_{3}\left[1-\frac{1}{1+exp[c_{1}(\;c_{2}-T)]}\right]$$

where *T* is the temperature, and $l_{1-3}$ and $c_{1-3}$ are the coefficients (Table 2).

A first-order electromagnetic scattering boundary condition was applied to the outer boundaries of the modeling domain of the microwave probe to limit the reflection of the outgoing electromagnetic waves.

(4) $$n\times \left(\nabla \times E\right)-jkn\times \left(E\times n\right)=0$$

where n represents the direction of propagation of the electromagnetic wave, and k indicates the wave number.

The boundary conditions of the inner and the outer conductors of the microwave antenna are the perfect electric conductor boundary conditions:

(5) n×E=0

The microwave source is modeled as a port boundary condition. The S-parameter port boundary conditions apply the port boundary condition at the inlet of the microwave antenna with the input powers set of 65 W and 50 W.

(6) $$S=\frac{\int \left(E-E_{1}\right)\cdot E_{1}}{\int E\cdot E_{1}}$$

where is E excitation plus the reflected field, and $E_{1}$ is the electric field of the port.

The initial temperature of the domain was set at the human body temperature. In addition, the thermal insulation boundary condition was selected at the outer simulation boundaries.

(7) $$n\cdot \left(k\nabla T\right)=0$$

Pennes bioheat equation was used to estimate the heat transfer in biological tissue and can be used to describe heat transfer during MWA [36].

(8) $$\rho C\frac{\partial T}{\partial t}=\nabla \cdot (k\nabla T)+\rho_{b}C_{b}\omega_{b}\left(T_{b}-T\right)+Q_{met}+Q_{ext}$$

where ρ, C, k, and T are the density (kg/m^3^), specific heat capacity (J/kg·K), thermal conductivity (W/m·K), and temperature (K) of tissue, respectively, and $\rho_{b}$, $C_{b}$, $\omega_{b}$, and $T_{b}$ are the density, specific heat capacity, perfusion (1/s), and temperature of blood, respectively. The metabolic heat source $(Q_{met}$) is 33,800 W/m^3^ [37,38], whereas the external heat source ($Q_{ext}$), which is equal to the resistive heat generated by the electromagnetic field, is defined as

(9) $$Q_{ext}=\rho \cdot SAR$$

The specific absorption rate (SAR) is widely used to quantify the rate at which tissue absorbs electromagnetic energy during MWA. The SAR represents the electromagnetic power deposited per unit mass in tissue (W/kg) and is defined as

(10) $$SAR=\frac{\sigma {\left|E\right|}^{2}}{2\rho }$$

Therefore, the external heat source can be defined as

(11) $$Q_{ext}=\frac{\sigma {\left|E\right|}^{2}}{2}$$

where σ is the electrical conductivity (S/m), and E is the electric field (V/m).

In this study, the thermal conductivity and blood perfusion rate were considered functions of the temperature and defined as [37,39]

(12) k(T)=0.001265×T+0.4882

(13) $$\omega_{b}(T)=0.000021\times T+0.00035$$

The electrical and thermal properties of liver tissue, tumor, and blood vessels used in this study [37,38] are presented in Table 3.

The tissue damage caused by MWA depends on the temperature and MWA duration.

In the context of MWA, Arrhenius modeling involves using mathematical equations to predict the thermal damage and coagulation of tissues due to raised temperatures. The Arrhenius tissue damage model was employed to determine the ablation zones. The degree of thermal damage was defined as

(14) $$\alpha \left(t\right)=\int_{0}^{t}A\;exp(-\frac{\Delta E}{RT(\tau )})d\tau$$

where $\alpha \left(t\right)$ is the degree of tissue damage over time, T(τ) is the temperature at time τ, A is the frequency factor (1/s), ΔE is the activation energy (J/mol), and *R* is the gas constant (J/K·mol); the Arrhenius kinetic parameters can be found in Table 4 [40,41,42]. In addition, the fraction of necrotic tissue ($\theta_{d}$) can be calculated by

(15) $$\theta_{d}=1-e^{-\alpha \left(t\right)}$$

where $\theta_{d}$ represents the percentage of cell death, with a threshold of $\theta_{d}$ > 0.98, indicating cell necrosis. This Arrhenius model threshold is used to quantify necrosis resulting from thermal damage [43,44,45,46,47].

### 2.4. Model Performance

Several metrics were calculated to assess the accuracy performance of the models, including the Dice score, which measures the overlap between the clinical ablation zones and those predicted by the patient-specific 3D and Vendor Prediction models; sensitivity, which describes the ability of the models to correctly predict the clinical ablation zone; specificity, which describes the capability of the models to exclude non-clinical areas; and mean distance to agreement (DTA), which quantifies the average agreement between clinical and predicted ablation zones.

## 3. Results

**Figure 3:**
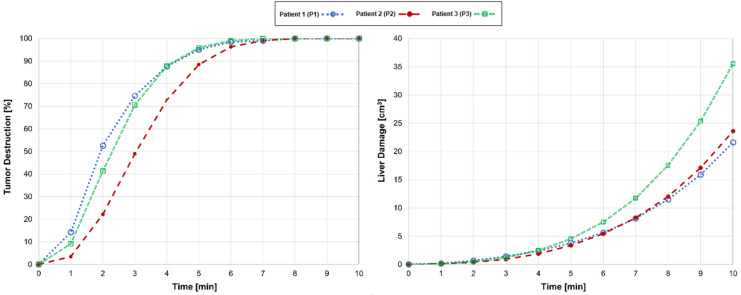
Comparative analyses of tumor destruction (**left**) and liver tissue damage (**right**) over a standard ablation time of 10 min for patient 1 (P1; blue dotted line), patient 2 (P2; red dashed line), and patient 3 (P3; green dashed line).

Comparisons of the tumor destruction and liver tissue damage for patients 1, 2, and 3 are shown in Figure 3. The ablation procedures were performed under the same clinical parameters; each patient employed an input power and a standard ablation time of 10 min. For patient 1 (blue dotted line), who underwent MWA with a clinical input power of 65 W, tumor destruction occurred rapidly, and the model predicted complete tumor destruction at 8 min; continuing the therapy beyond this point only damaged the healthy tissue surrounding the tumor. Compared with stopping at 10 min, stopping at 8 min, upon total tumor destruction, reduced tissue damage by 50% while consistently achieving the 5 mm MAM.

Patient 2 (red dashed line), whose tumor was approximately 44% larger than patient 1, complete tumor destruction at 8 min happened with an input power of 50 W. Terminating at this point led to a 46% reduction in tissue damage while maintaining the 5 mm MAM. Patient 3 (green dashed line), whose tumor was larger than patients 1 and 2, experienced total tumor destruction at 7 min achieved with an input power of 65 W. Stopping at this time reduced tissue damage by 66% compared to the standard ablation time while consistently achieving the 5 mm MAM. Overall, these observations indicate that stopping MWA at the optimal time minimizes tissue damage while ensuring the efficacy of the treatment and achieving the 5 mm MAM.

**Figure 4:**
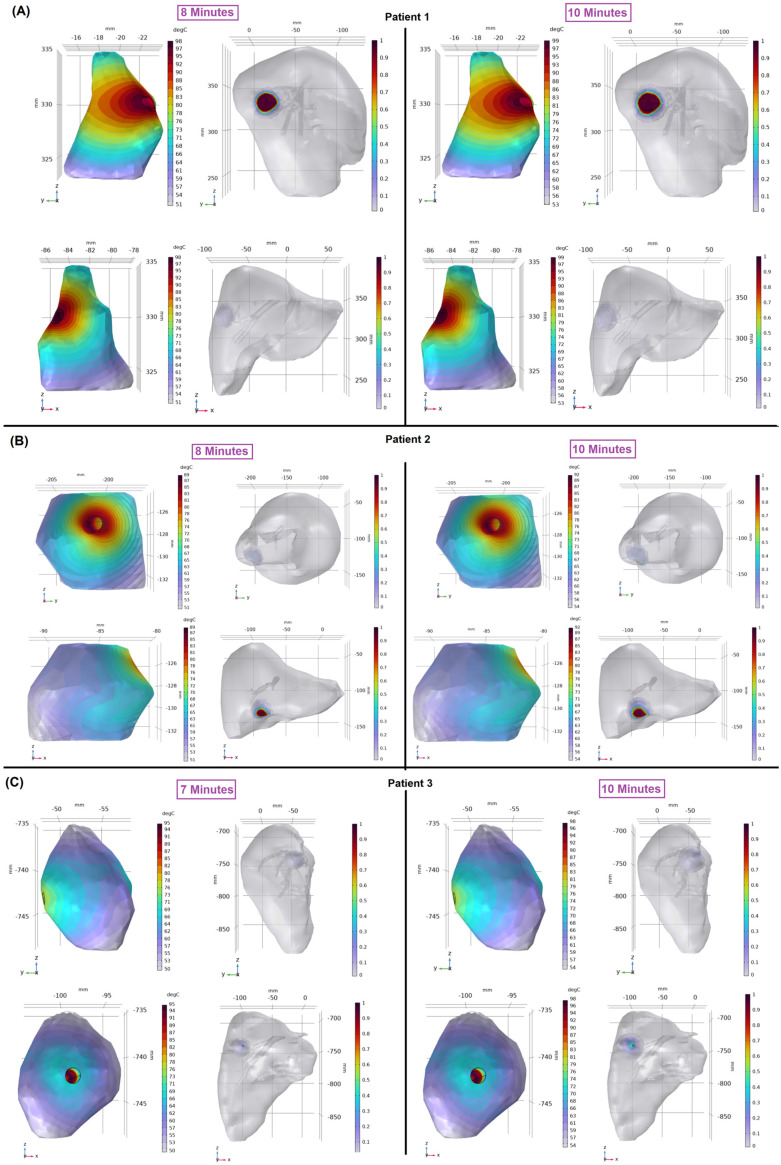
Temperature distribution in the tumor (left; color bars indicate temperatures in degrees Celsius) and liver damage fraction (right; ablation volume captures regions where the Arrhenius value $\theta_{d}$ > 0.98) for patient 1 (**A**), patient 2 (**B**), and patient 3 (**C**). For each patient, the coronal view (**top**) and sagittal view (**bottom**) are shown.

The temperature distributions within the patients’ tumors are shown in Figure 4 to provide additional insights into tumor destruction and liver damage. For patient 1 (Figure 4A), the temperature within the tumor exceeded the critical threshold of 50 °C, which is known to destroy cancer cells effectively [48,49] at 8 min. Stopping the therapy at this time maintained treatment effectiveness while minimizing damage to the surrounding healthy liver tissue. However, continuing the MWA therapy until the end of the ablation time resulted in an expanded ablation zone and irreversible damage to healthy tissue (Figure 4A, right). For patient 2 (Figure 4B), the tumor temperature exceeded 50 °C at 8 min. Terminating the MWA at this point retained treatment efficacy and reduced damage to healthy liver tissue. In patient 3 (Figure 4C), the temperature reached the critical level at 7 min. For all three patients, the prolonged ablation time enlarged the ablation zone and irreversibly damaged the surrounding healthy tissue without having a substantial impact on accomplishing complete tumor destruction, especially when both complete tumor destruction and adequate MAM were achieved earlier.

**Figure 5:**
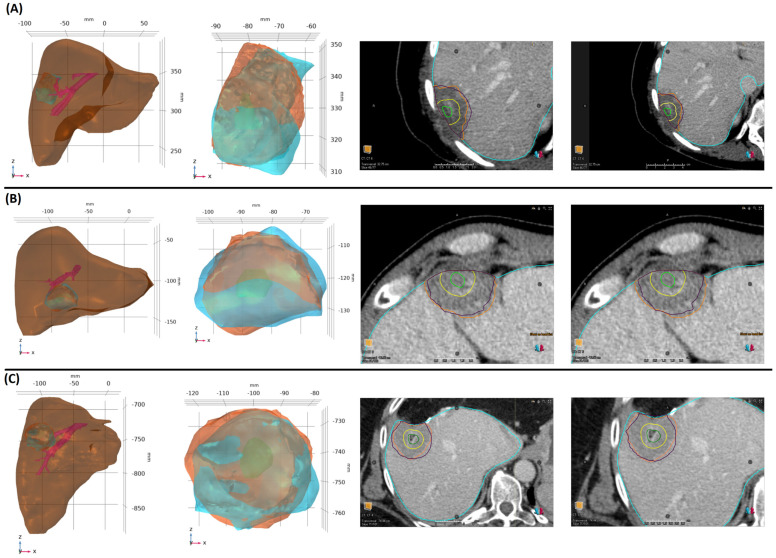
Patient-specific 3D models for patient 1 (**A**), patient 2 (**B**), and patient 3 (**C**). In each panel, the first image from the left shows the liver, tumor, and blood vessels, and the second image shows the clinical ablation zone (blue), computational ablation zone (orange), and tumor (green). The third and fourth images are post-ablation CT images showing the liver (blue), tumor (green), 5 mm MAM (yellow), clinical ablation zone (orange), and predicted ablation zone (purple) for ablation times of 10 min and 9.5 min, respectively.

Images of 3D models for all three cases clearly show the overlap of the clinical and predicted ablation zones and help validate the accuracy of the model (Figure 5). In each panel, four sets of images are provided. The left column displays comprehensive 3D patient-specific models, including the liver, tumor, blood vessels, clinical, and predicted ablation zones. The second column from the left represents the tumor (green), highlighting the clinical (blue) and predicted (orange) ablation zones. The third column from the left and the last column show post-ablation CT images mapping the tumor (green), 5 mm MAM (yellow), clinical (orange), and predicted (purple) ablation zones for ablation times of 10 min and 9.5 min, respectively. The performance metrics and the clinical and predicted MAMs for each of the three patients, considering the approximate end of the ablation time (10 min) and earlier uncertainty estimate values (9.5 min) for the predicted ablation zones, are presented in Table 5.

For patient 1, the 3D model demonstrated significant overlap between clinical and predicted ablation zones at 10 and 9.5 min, with Dice scores of 0.73 and 0.77, respectively. The sensitivity remained consistent, the specificity improved from 0.76 to 0.85, and the mean DTA slightly reduced from 2.6 mm to 2.3 mm. The Vendor Prediction showed a lower Dice score and sensitivity of 0.7 and 0.63, with a specificity of 0.88 and a mean DTA of 2.6 mm. For patient 2, the 3D model achieved high accuracy, with Dice scores of 0.86 and 0.83 for ablation times of 10 and 9.5 min, respectively. The sensitivity was 0.79 and 0.72, with a consistently high specificity of 0.96 and 0.98. The mean DTA remained at 1.2 mm for both times. In comparison, the Vendor Prediction had a significantly lower accuracy, with a Dice score of 0.54, sensitivity of 0.38, specificity of 0.98, and a much larger mean DTA of 4.3 mm. For patient 3, the 3D model preserved high accuracy with Dice scores of 0.8 for both ablation times, sensitivity values of 0.85 and 0.79, and specificity values of 0.74 and 0.82. The mean DTA was 2.5 mm for both times. The Vendor Prediction had lower Dice scores and sensitivity values of 0.7 and 0.58, respectively, at 10 min, with a specificity of 0.91 and a larger mean DTA of 3.7 mm.

These results highlight the effectiveness of patient-specific 3D models in predicting MWA outcomes. To assess this efficacy, the predicted ablation zones from the 3D models with the actual ablation zones observed in clinic were compared and revealed with the higher Dice scores, sensitivities, and specificities, indicating a closer alignment between the clinical and predicted ablation zones. The mean DTA values confirm the overall agreement, highlighting the effectiveness of 3D models in predicting accurate ablation zones, in contrast to the lower performance of the Vendor Prediction.

## 4. Discussion

MWA is a promising treatment in interventional oncology for up to 80% of ineligible patients for liver resection [50]. Tumor recurrence remains a significant issue despite treatment effectiveness, with a five-year survival rate of up to 52%. Incomplete tumor destruction and inadequate MAM are consistently identified as the primary factors contributing to local tumor recurrence [22,23,51,52]. A recent study [53] highlighted that thermal ablation may change the interstitial space of tissue, affecting the heating requirements of tumors based on tumor vasculature. They suggested that thermal damage results in the release of intercellular fluid after the rupture of cell membranes, consequently increasing the interstitial volume fraction or porosity. Such changes in the interstitial structure enhance porosity, which can affect both the heat distribution during ablation and the diffusion properties within the tumor. In addition, it is hypothesized that local heating may increase blood flow and promote lymphatic drainage, further altering the interstitial space structure. These alterations suggest that the thermal dosage, if sufficient to cause irreversible damage, might lead to significant changes in the tumor porosity, impacting the efficacy of the ablation process.

A comprehensive MWA planning system incorporating patient-specific parameters for pre-therapy assessments is absent. The lack of these specific planning tools can prevent physicians from gaining in-depth insights into treatment outcomes. This gap may cause an increase in the number of re-ablation cycles to achieve complete tumor destruction and a safe MAM, thereby raising the risk of over-ablation, which can induce irreversible damage to healthy tissue. To address this challenge, researchers have performed computational studies integrating all necessary parameters to help predict and estimate MWA treatment outcomes. Although fully 3D models are complex and computationally demanding, most numerical studies rely on 2D axisymmetric models [48,54,55]. Existing 3D simulation investigations do not account for heterogeneity, tumor morphology, variations in tumor location, realistic microwave antenna insertion paths, or real blood vessels [56,57]. Therefore, developing a robust, patient-specific 3D planning system is crucial to providing the insights and data necessary for well-informed decisions and ultimately advancing the accuracy and safety of MWA procedures.

Tumor responses to MWA are complex; factors like the size and number of tumors can influence the likelihood of recurrence following MWA [58,59,60,61]. Thus, patient parameters must be considered to enhance treatment outcomes. Considering the complex tumor topology details, the importance of comprehensive 3D models and simulations is well established [56,57,62]. Moreover, employing a high input power and short ablation time can result in elongated ablation zones, potentially causing unintended damage to the healthy tissue surrounding the tumor [62,63,64]. Several studies have shown that microwave propagation can influence the degree of thermal damage and the ablation zone shape. This process depends on various factors, including the input power, ablation time, antenna type, microwave frequency, and distance from the antenna [38,49,65]. Our findings align with this existing research, suggesting that relying solely on axisymmetric investigations and ex vivo models may not accurately determine the most suitable input power and ablation time. There is an inherent inconsistency in how MWA interacts with tissue, leading to irregular ablation zones. This variability can contribute to under- and overtreatment, which is a significant concern for achieving optimal therapeutic outcomes. A study [66] demonstrated this discrepancy, showing that the ablation zone predictions provided by vendors often do not align with the actual ablation zones observed in clinical practice. Our results emphasize the limitations of relying on the vendor information to predict an ablation zone, revealing distinctions between the predicted ablation zones from the Vendor Prediction and the clinical outcomes. These significant inconsistencies highlight the potential for under- or overtreatment and the need for more reliable and patient-specific predictive models to guide MWA therapy and optimize treatment outcomes. Therefore, it is critical to incorporate 3D models for a more comprehensive evaluation and to improve therapy outcomes based on patient characteristics.

This study simulated MWA under similar clinical parameters, including input power, microwave antenna insertion, and a standard ablation time for three patients with distinct tumor, liver, and vasculature anatomical characteristics and positioning. The findings emphasize the importance of considering patient-specific anatomical features and the potential for optimizing treatment to enhance the efficacy and minimize the damage to healthy tissues. In the simulated MWA procedures for the three patients, our analysis suggests that the earlier termination of ablation could significantly reduce liver damage while maintaining effective therapeutic outcomes. Patient 1, stopping the MWA procedure at 8 min instead of the standard 10 min, reduced liver damage by approximately 50%. Patient 2, ending the ablation at 8 min, resulted in a 46% decrease in liver damage. For patient 3, finishing the treatment at 7 min achieved a reduction in liver damage by about 66% while still ensuring complete tumor destruction and maintaining the necessary 5 mm MAM for all three patients.

These simulation results demonstrate that adjusting the ablation time based on real-time monitoring of tumor responses could allow for a more tailored approach, minimizing the unnecessary damage to healthy liver tissue and reducing potential complications. This approach highlights the value of patient-specific simulation in optimizing MWA. Patient 1 experienced liver damage of about 22 cm^3^, with a small tumor size of 0.43 cm^3^ located very close to the liver surface (0.1 mm) and near to major blood vessels (12.4 mm); the observed damage was considerable, given the minimal tumor volume and closeness to critical structures. Patient 2 had slightly more liver damage, about 24 cm^3^, despite having a larger tumor size of 0.62 cm^3^; the tumor was located 0.2 mm from the liver surface and a farther distance from blood vessels (24.4 mm). The larger liver volume (5547 cm^3^) and bigger tumor size compared to patient 1 could factor into the slightly increased damage. Patient 3 indicated the most substantial liver damage, about 35 cm^3^. The largest tumor (0.88 cm^3^) was situated deeper within the liver (9.1 mm from the surface) and closer to major blood vessels (19 mm); the combination of large tumor size and its more in-depth location could potentially contribute to more significant liver damage.

Achieving a 5–10 mm MAM ensures the long-term success of MWA treatment and reduces the risk of local tumor progression and recurrence [17,22,23,67]. In the present study, a MAM of 5 mm was achieved clinically and computationally for all three patients, regardless of whether the therapy was completed at the scheduled ablation time (9.5 or 10 min) or the optimized ablation time (8 min for patients 1 and 2 and 7 min for patient 3). This consistent achievement of the MAM is reassuring, as it aligns with the goal of preserving healthy tissue while destroying the tumor completely.

Stopping MWA at the optimal time ensures complete tumor destruction and the achievement of MAM, preserving healthy tissue and organ function. This approach is especially beneficial for patients with multiple tumors, recurrent tumors, or those requiring the following treatments, prioritizing patient-centric outcomes. This finding highlights the necessity of a safe MAM for optimal MWA termination, balancing the ablation duration with MAM achievement to avoid damage to healthy tissue and enhance therapy success. The effectiveness of patient-specific 3D models in MWA outcome prediction is demonstrated through high Dice scores, sensitivities, and specificities, indicating a strong alignment between the clinical and predicted ablation zones. The similar mean DTA values further support the accuracy of the model in guiding MWA. These findings are crucial for assessing the suitability of the model for clinical application.

Uncertainties and unknown parameters remain significant challenges in MWA therapy, particularly in the absence of a patient-specific treatment planning system. In addition, there is a lack of information regarding the accurate ablation time and whether the microwave antenna was removed immediately or left in place until it cools. By accounting for these uncertainties, our patient-specific 3D model demonstrates reasonable simulated outcomes that closely align with clinical results. Recording these parameters during treatment is critical to enabling future studies on larger cohorts of patients. Our findings highlight the potential for more precise, patient-centered treatments that optimize the ablation times and minimize damage to healthy liver tissue. Further investigations are needed to explore the broader applicability of patient-specific 3D modeling and to determine how other factors, such as antenna insertion paths, antenna type, biological parameters, and tumor location, influence MWA treatment outcomes.

This study provides valuable insights into applying patient-specific 3D models for the MWA treatment of liver tumors. However, it is essential to acknowledge some limitations. This study employed the Pennes bioheat equation with homogeneous blood perfusion, varying linearly as a function of temperature, which may not fully capture the clinical complexities of tumor ablation. A recent study by Singh [68] suggested the importance of considering heterogeneous blood perfusion in tumor regions to enhance ablation effectiveness. In addition, a recent study [69] reviewed various transfer models for human tissue and reported alternative models, such as those by Wulff [70], Klinger [71], Chen and Holmes [72], and Nakayama and Kuwahara [73], who introduced enhancements by modeling blood flow with directional convection terms, considering larger vessels separately, or addressing local thermal non-equilibrium conditions; these models posed significant challenges in terms of data availability and complexity of implementation. For instance, the models proposed by Chen and Holmes and Nakayama and Kuwahara necessitate an in-depth knowledge of the vascular network and complicated implementation procedures, which could limit their practical application in studies like ours, where such detailed data still need to be included. The Pennes equation, despite its limitations, continues to be widely used due to its feasibility in scenarios where data on detailed vascular anatomy are unavailable. However, acknowledging the potential for enhanced precision with more complex models, we aim to explore these advanced bioheat transfer models in future studies. This approach will enable us to incrementally refine our simulation capabilities, particularly in more complex clinical scenarios where the directionality of blood flow and detailed vascular anatomy play critical roles in the outcomes of thermal therapies.

The small cohort was specifically selected based on the following criteria: all patients were treated using a single MWA antenna insertion without displacement and with consistent MWA device settings throughout their therapy. These conditions were necessary to maintain controlled variables for accurately validating the predictive capabilities of 3D models against clinical outcomes and comparing these with the Vendor Prediction model. The strict criteria were necessary for this initial validation study; however, the design of future studies must include the flexibility to predict the ablation in the diverse configurations of the range of patient characteristics seen clinically. In addition, the study was performed using a single vendor MWA device; future studies are required to evaluate how these results predict the outcomes from other systems. Future research should also include assessments of the changing physiological parameters and expand the study to include more patients, thus enhancing the robustness and clinical relevance of the 3D modeling approach.

## 5. Conclusions

This study underscores the impact of tumor size and location on determining the optimal ablation time and power settings. For example, in our computational model, the patient 1 predicted Dice Score was 0.73, which was approximately 4.3% higher than the vendor model at 0.7, with a sensitivity improvement from 0.63 to 0.72, an increase of about 14.3%. For patient 2, the predicted Dice Score was substantially better at 0.86, compared to the vendor model at 0.54, marking a significant 59.3% improvement. The sensitivity for this patient improved dramatically from 0.38 to 0.79, showing a 107.9% increase. Patient 3 also demonstrated enhanced outcomes, with our model predicting a Dice Score of 0.8 compared to the vendor model at 0.7, a 14.3% increase, and the sensitivity improving from 0.58 to 0.85, a 46.6% improvement. These results emphasize the precision of the 3D model and demonstrate significant variability in the thermal ablation efficacy based on patient-specific anatomical factors. The absence of blood flow modeling in our current framework points to a critical area for future enhancements. By incorporating a dynamic blood flow, we could significantly refine our understanding of these variations, enabling even more precise and patient-specific ablation therapies.

In summary, the findings highlight the significant potential of employing patient-specific 3D models in improving the precision and effectiveness of MWA treatments for liver tumors. The results demonstrate that 3D models can accurately predict ablation zones, align closely with clinical outcomes, and have higher accuracy than Vendor Prediction models and, therefore, have the potential to improve treatment efficacy. By integrating detailed patient characteristics and leveraging computational simulations, these models allow for more targeted ablation strategies, reducing the risk of recurrence and unnecessary damage to surrounding healthy tissues. Moreover, further research is needed, particularly in refining these models to address the uncertainties surrounding the optimal ablation times, input power, and antenna placement management. Ultimately, it provides patient-centered MWA therapies, enhancing outcomes and offering a higher standard of patient care.

## Figures and Tables

**Table 1:** Patient demographic and characteristics.

	Age	Sex	Tumor Histology	Specify Histology	Tumor Location	Tumor Size [cm^3^]	Liver Size [cm^3^]	Blood Vessels Size [cm^3^]
**P1**	41	Female	Non-colorectal	Breast cancer liver metastasis	Right lobe (Segment 5–8)	0.43	1288	7.3
**P2**	51	Male	Colorectal	-	Left medial (Segment 4)	0.62	5547	2.1
**P3**	64	Female	Non-colorectal	Cholangiocarcinoma	Right lobe (Segment 5–8)	0.88	1310	8.9

**Table 2:** The coefficients of the temperature-dependent electrical properties [34,35].

	Liver			Tumor		
	$l_{1}$	$l_{2}$	$l_{3}$	$c_{1}$	$c_{2}$	$c_{3}$
Relative permittivity ($\varepsilon_{r}$)	0.076	82.271	48.391	5.223	0.052	54.8
Electrical conductivity (σ)	0.069	85.375	2.173	6.583	0.059	2

**Table 3:** Electrical and thermal properties of healthy liver tissue, tumor, and blood vessels.

Tissue	Electrical Conductivity (S/m)	Relative Permittivity	Thermal Conductivity (W/m·K)	Specific Heat (J/kg·K)	Density (kg/m^3^)
**Liver**	Equation (3)	Equation (2)	Equation (9)	3540	1079
**Tumor**	Equation (3)	Equation (2)	Equation (9)	3960	1040
**Blood vessels**	2.54	58.3	0.5	3600	1060

**Table 4:** Arrhenius kinetic parameters used in this study [40,41,42].

Tissue	Activation Energy [J/mol]	Frequency Factor [1/s]
Liver	2.58 × 10^5^	7.39 × 10^39^
Tumor	2.81 × 10^5^	3.25 × 10^4^

**Table 5:** Performance metrics for patient-specific 3D models of MWA accuracy. Values are bolded in the table to highlight the higher predictive performance metrics of the patient-specific 3D models over vendor predictions.

	Ablation Time (min)	Dice Score		Sensitivity		Specificity		Mean DTA (mm)	
		3D Model	Vendor Prediction	3D Model	Vendor Prediction	3D Model	Vendor Prediction	3D Model	Vendor Prediction
**P1**	10	0.73	0.7	**0.72**	0.63	0.76	**0.88**	2.6	2.6
	9.5	**0.77**	-	**0.72**	-	0.85	-	**2.3**	-
**P2**	10	**0.86**	0.54	**0.79**	0.38	0.96	**0.98**	**1.2**	4.3
	9.5	0.83	-	0.72	-	**0.98**	-	**1.2**	-
**P3**	10	**0.8**	0.7	**0.85**	0.58	0.74	**0.91**	**2.5**	3.7
	9.5	**0.8**	-	0.79	-	0.82	-	**2.5**	-

## Data Availability

The data generated or analyzed during the study are available from the corresponding author by request.
